# An Overview of the Influence of Breastfeeding on the Development of Inflammatory Bowel Disease

**DOI:** 10.3390/nu15245103

**Published:** 2023-12-13

**Authors:** Benjamin Bertin, Benoit Foligne, Delphine Ley, Jean Lesage, Laurent Beghin, Jules Morcel, Frédéric Gottrand, Emmanuel Hermann

**Affiliations:** 1 Univ. Lille, Inserm, CHU Lille, U1286-INFINITE-Institute for Translational Research in Inflammation, F-59000 Lille, France; benjamin.bertin@univ-lille.fr (B.B.); benoit.foligne@univ-lille.fr (B.F.); delphine.ley@chu-lille.fr (D.L.); jean.lesage@univ-lille.fr (J.L.); laurent.beghin@chu-lille.fr (L.B.); jules.morcel.etu@univ-lille.fr (J.M.); frederic.gottrand@chu-lille.fr (F.G.); 2 Univ. Lille, Inserm, CHU Lille, CIC-1403 Inserm-CHU, F-59000 Lille, France

**Keywords:** early life, breastfeeding, milk, microbiota, immune system, inflammatory bowel diseases

## Abstract

The first 1000 days of life is a critical period that contributes significantly to the programming of an individual’s future health. Among the many changes that occur during this period early in life, there is growing evidence that the establishment of healthy gut microbiota plays an important role in the prevention of both short- and long-term health problems. Numerous publications suggest that the quality of the gut microbiota colonisation depends on several dietary factors, including breastfeeding. In this respect, a relationship between breastfeeding and the risk of inflammatory bowel disease (IBD) has been suggested. IBDs are chronic intestinal diseases, and perinatal factors may be partly responsible for their onset. We review the existence of links between breastfeeding and IBD based on experimental and clinical studies. Overall, despite encouraging experimental data in rodents, the association between breastfeeding and the development of IBD remains controversial in humans, partly due to the considerable heterogeneity between clinical studies. The duration of exclusive breastfeeding is probably decisive for its lasting effect on IBD. Thus, specific improvements in our knowledge could support dietary interventions targeting the gut microbiome, such as the early use of prebiotics, probiotics or postbiotics, in order to prevent the disease.

## 1. Introduction

The risk of chronic disease in adulthood is associated with environmental events during perinatal life and early childhood in a period known as the first 1000 days of life, from conception to the age of two years. According to this paradigm, environmental factors and dietary habits early in life are determinants of individual development and subsequent health, particularly for non-communicable diseases. Since Barker’s first observations in the late 1980s, the early postnatal period has been shown to be associated with the risk of long-term cardiovascular diseases [1]. Epidemiological studies have subsequently confirmed Barker’s work and suggested a role for the early environment in the occurrence of neurological, metabolic or cardiovascular disorders later in life [2,3,4,5,6]. The food restrictions during the 1944 famine in the Netherlands led to an increase in chronic pathologies, including further obesity among the generations born at that time [7], with persistent effects for the following generations [8]. Consequently, this work also highlights the fact that maternal malnutrition during gestation impedes the normal development of placentation, with subsequent consequences for the risk of chronic degenerative disorders [9] or inflammatory bowel disease (IBD) [10].

All these prior observations were of growing interest to the scientific community and led to the paradigm of the developmental origin of health and disease (DOHaD) [11,12]. Epigenetics, which modulates the expressions of genes without modifying their sequences, is one of the biological components of the calibration and perpetuation of early environmental events that influence an individual’s health [13,14]. For instance, genetic inheritance and/or epigenetics can partly predict risks of metabolic disorders [14]. The microbes that colonise the neonatal gut immediately following birth and shape the host immunity [15] are able to regulate the chemical phenomena of histone acetylation and DNA methylation via the metabolites it produces, such as short-chain fatty acids (SCFAs) [16]. Breastfeeding by modulating the development of the child’s microbiota could also participate in epigenetic modifications [17,18,19].

Early parent–child interactions, educational factors (sleep, exposure to screens), the parental lifestyle (diet, exposure to psycho-social stressors, physical activity) and exposure to toxic substances are all environmental factors with likelihoods of leaving lasting imprints on a child’s health [5,20,21,22]. Environmental stressors, including exposure to environmental xenobiotics and poor nutritional status, like inadequate fat or carbohydrate intake, can have multiple consequences for placental functions, with consequences for future health [23,24]. Other epidemiological studies in humans have highlighted the many perinatal factors, such as the mode of delivery, type of infant feeding, antibiotic therapy or tobacco exposure during the first months of life, which can have determining influences on the subsequent risk of chronic intestinal diseases, such as celiac disease or IBD, including ulcerative colitis (UC) and Crohn’s disease (CD) [25].

## 2. Breastfeeding

### 2.1. General

Exclusive breastfeeding for at least the first 6 months is the benchmark for optimal infant growth [26]. This recommendation is based on evidence that the composition of breastmilk and its energy intake are perfectly suited to the child’s needs [27,28], with beneficial effects depending on the duration of breastfeeding and the age of complementary feedings [29]. The most obvious benefits of breastfeeding include neurodevelopment in preterm infants and the prevention of respiratory and gastrointestinal infections and allergies in children [30,31]. It is also well known that breastfed preterm infants present a lower risk of necrotising enterocolitis (NEC) [32]. As an example, the PROBIT (Promotion of Breastfeeding Intervention Trial) interventional study, previously implemented in Byelorussia, which was specifically aimed at promoting breastfeeding, showed a health benefit by decreasing the risk of gastrointestinal-tract infections and atopic eczema at one year of age, but with no change in the prevalence of respiratory-tract infection [33]. However, while the positive influence of breastfeeding seems to be most evident in low-income countries, a more moderate effect is observed in developed countries where health and social security are better developed [31]. Furthermore, a relationship between breastfeeding and the risk of long-term health outcomes has also been widely emphasised, with sometimes contradictory findings, showing, in particular, a likely effect of breastfeeding on reducing early adiposity rebound, obesity and type 2 diabetes [31,34]. These observations are supported by several works that have suggested that the early disruption of the gut microbiota increases the propensity for later metabolic deregulation [35]. These vulnerabilities manifest as long-lasting endocrine, metabolic and inflammatory effects on the offspring [6]. Breastfeeding has been involved in the protection against various immune-mediated diseases [36], although this is still a matter of debate [31].

### 2.2. Immune and Gut Microbiota Maturation

**Figure 1:**
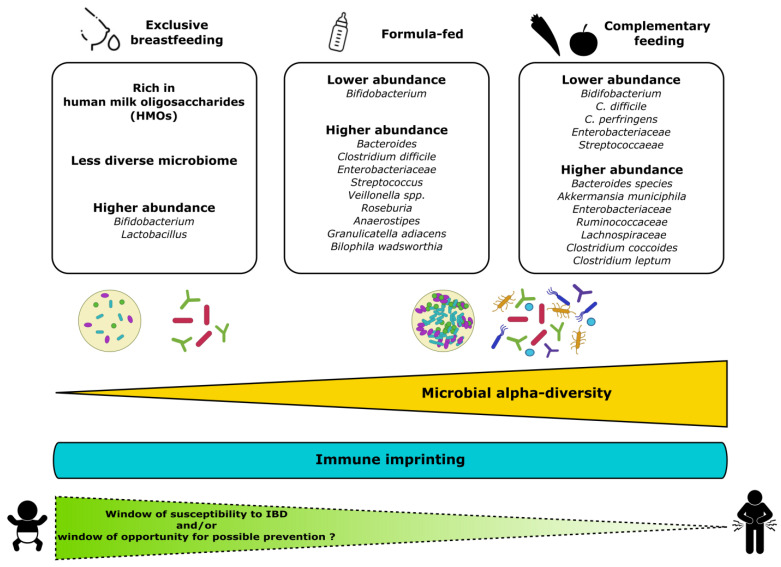
Composition of gut microbiota in early life in relation to child’s diet. *Bifidobacterium* predominates in exclusively breastfed infants, while, in formulae-fed infants, the composition is less uniform and notably enriched with *Bacteroides*, *Streptococcus* or *Clostridium*. The introduction of solid foods leads to a wider range of microorganisms with greater microbial α-diversity and abundance. The establishment of interactions between the host immunity and the microbiota may result in susceptibility to or protection against the onset of IBD later in life. It is relevant to consider the first months of life as a window of opportunity for preventive dietary intervention to promote early protective effects.

The epithelial barrier and microbiota together contribute to immune homeostasis and the acquisition of tolerance to commensal bacteria and dietary antigens during the early postnatal period. Numerous studies indicate that early feeding, and particularly breastmilk, influences the development of the gut barrier and microbiota colonisation and enhances the maturation of the immune system [27,37]. The beneficial influence of breastmilk can be directly attributed to its bioactive components (macronutrients and micronutrients, oligosaccharides, immunoglobulins, cytokines, leukocytes as well as viable microbiota) [37,38]. In particular, lactoferrin is an iron-binding protein that plays an important role in protection against microbial infections. Lactoferrin also exhibits properties that modulate the host immune defence in the intestine [39]. Moreover, the microbiota in human milk may play a defensive role against gastrointestinal infections by participating in the early colonisation of the gut in newborns, contributing to the maturation of the immune system [39]. Interestingly, studies have unravelled the immune development driven by gut microbiota in newborns and its postnatal adaptation to environmental insults [40,41]. In this vein, it has been suggested that the duration of breastfeeding has a greater impact on the intestinal microbial diversity of infants born via caesarean section than that of infants born vaginally [42]. The role of breastfeeding on the immunological status of the child is actually evident in the first months of life [38]: the production of secretory immunoglobulin A (sIgA), detectable in the stool, is increased early in life in breastfed children compared to children receiving infant formula [43,44]. sIgA is involved in intestinal homeostasis by regulating the expressions of genes involved in inflammation, modulating the diversity of the gut microbiota and protecting against infections [45,46,47]. The gut microbiota in early life undergoes a progressive increase in α-diversity and is shaped mainly by the child’s diet, as shown in Figure 1 [48,49,50,51,52,53,54]. In fact, the composition of the gut microbiota differs significantly between breastfed infants and those receiving infant formula (higher proportions of *bifidobacteria* and *lactobacilli*, which are overall beneficial for health in breastfed infants) [15,55,56]. Otherwise, in formula-fed infants, the gut microbiome is usually dominated by an increased abundance of *Enterococcus* or *Streptococcus* [54,57]. The cessation of breastfeeding, more than the introduction of solid foods, is the main driver in the dynamics of microbiota development during the first year of life [52,58]. The impact of the weaning stage on microbiota development has been poorly investigated but is thought to contribute to gut microbiota alpha diversity [15]. At weaning, increased abundances of the adult-type microorganisms *Bacteroides*, *Prevotella*, *Clostridium*, *Ruminococcaceae* or *Veillonella* occur, with decreases in *Bifidobacterium*, *Enterobacteriaceae* and *Streptococcaeae* [37,59]. Surprisingly, other studies indicate that the alpha diversity was even lower in preschoolers than in adults, but the long-term influence of early dietary habits on the transition to a mature microbiota in children remains poorly characterised [60]. A growing body of literature points to changes in the gut microbiota as the source of an early immune imprint that may influence long-term health [40,61].

Human milk is composed of diverse non-digestible oligosaccharides (human milk oligosaccharides (HMOs)) that enable the early growth of *bifidobacteria*, which encode HMO-utilising genes and are predominant during the first months of life [62]. By metabolising HMOs, *bifidobacteria* promote the release of SCFAs, which improve the epithelial barrier integrity or immune regulatory response by reducing Th2 and Th17 cytokines through interaction with G-protein-coupled receptors, such as GPR43, GPR41 and GPR109A expressed by epithelial and immune cells [40,63]. Beyond this, recent studies using selected HMOs in adult mice have shown that these prebiotics are able to reduce fat mass development, insulin resistance and hepatic steatosis [64,65], suggesting a therapeutic application of HMOs against the metabolic syndrome through the probable involvement of the release of numerous specific microbial metabolites.

Moreover, recent data have demonstrated that microbial metabolites largely mediate the impact of the microbiome on the host physiology [66,67]. Most of the metabolites generated by microbiota metabolism (e.g., SCFAs, such as acetate, propionate and butyrate, or other common metabolites, such as trimethylamine N-oxide (TMAO) or tryptophan derivates) may play a role in the induction of immune tolerance, the intestinal barrier function, signalling or epigenetic modulation that can determine the increased likelihood of developing immune-mediated diseases and systemic effects on health [27,68,69]. For instance, breastfeeding may promote *Bifidobacterium* species to convert tryptophane into metabolite derivatives, notably kynurenine and indole, which activate the aryl-hydrocarbon receptor (AhR). The AhR is associated with the expansion of type 3 innate lymphocytes (ILC3) and IL-22 production and regulates regulatory T-cell differentiation [70,71,72]. In turn, IL-22 signalling may influence the composition and function of the gut microbiota [72]. It is worth noting that a reduction in AhR ligand production and, consequently, IL-22 activation has been observed in IBD patients [73]. Although research in this field is presently sparse, this converging evidence suggests that microbial-derived metabolites can strongly influence developmental programming in breastfed infants [67]. Moreover, it can also be postulated that these compounds may also have potential impacts on intestinal and metabolic health as new “postbiotic” therapeutics to treat microbiome-related non-communicable diseases (NCDs) in infants and adults.

## 3. Breastfeeding and Risk of IBD

IBDs are chronic intestinal diseases, and perinatal factors may be partly responsible for their onset, although there is little evidence to suggest this [74]. Given that human milk can shape the gut immune response and microbiota with long-term benefits against immune-related diseases [36], the role of breastfeeding on the subsequent risk of CD and UC has been extensively examined. We propose to review the existence of a link between breastfeeding and IBD from experimental and clinical studies.

### 3.1. IBD Presentation

CD and UC are the two main clinical forms of IBD. Defined empirically on the basis of clinical, endoscopic and radiological criteria, they are characterised by the chronic and recurrent inflammation of the intestinal wall. Although the exact origin of IBD remains unknown, the current hypothesis is that it is a complex, multifactorial disease, occurring in genetically predisposed individuals and resulting in an abnormal mucosal immune response to intestinal microflora [75]. Over the past 20 years, more than 200 susceptibility genes associated with IBD have been identified [76,77,78]. To date, only smoking and appendectomy are environmental factors recognised as being linked to IBD, even if their mechanisms have not yet been clarified. The impact of current smoking on the IBD course has been studied extensively; smoking is deleterious in CD and beneficial in UC [75,79].

Of note, the incidence and prevalence of IBD, and particularly in paediatric onset, are increasing, with a key role played by environmental risk factors [80,81]. In detail, the epidemiology of IBD is evolving steadily worldwide: the prevalence continues to rise in Western countries (Europe, North America), reaching over 0.3%, while the incidence is increasing rapidly in newly industrialised countries in Africa, Asia and South America [82]. Particular attention needs to be paid to the increase in IBDs in children and adolescents because of the impact that these diseases can have on their quality of life, such as stunted growth, school absenteeism and the psychological effect of a chronic disease on the patient and family [80]. Except for enteral nutrition, there are only limited data regarding the impact of diet on the disease course either considering adults [83,84] or children [85]. It should be noted that there is growing evidence of the role of the Western diet in the increasing prevalence of IBD worldwide [75,82,86].

### 3.2. Milk Components and Gut Inflammation: What Does an Experimental Model of Colitis Tell Us?

Over the last 30 years, numerous experimental models of colitis have been developed in rodents to decipher the underlying mechanisms of the IBD pathophysiology, identify molecular targets and evaluate new therapeutic strategies [87]. Among these different models of colitis, the most widespread are those induced by chemical compounds such as dextran sulphate sodium (DSS) or 2,4,6-trinitrobenzene sulphonic acid (TNBS), which are reputed to have many similarities with human UC and CD, respectively [88,89]. Genetic models built on the basis of susceptibility genes identified in IBD are also available but are less frequently used [90]. The potentially beneficial effects of breastmilk in these experimental models of gut inflammation have been tested by various teams, with a particular focus on milk-derived oligosaccharides and extracellular vesicles (EVs). In an initial study in 2002, Madsen et al. used interleukin-10-deficient mice, which developed spontaneous colitis, to study the role of breastfeeding on the progression of intestinal inflammation. They observed that breastfeeding had a beneficial effect on reducing the histological inflammation of the colon, as well as the circulating levels of TNF and IFNγ [91]. Subsequently, it was demonstrated that a rodent diet enriched with goat’s milk oligosaccharides (GMOs), administered in a preventive manner seven days before the induction of colitis, was able to reduce the acute intestinal inflammation induced by DSS in rats [92]. In control animals that did not receive DSS, the GMO diet caused a modification of the colonic microbiota with an enrichment in *lactobacilli* and *bifidobacteria*. At the same time, the preventive and anti-inflammatory effect of GMOs was also demonstrated in a TNBS rat model [93]. Fuhrer et al. used a different and original approach to investigate the role of the sialylated milk oligosaccharides in mucosal immunity [94]. In order to identify the respective roles of α2,3-sialyllactose (3′-SL) and α2,6-sialyllactose (6′-SL) on gut immunity, these authors used 2,3- and 2,6-sialylltransferase-deficient mice (St3gal4^−/−^ and St6gal1^−/−^ mice, respectively) and applied a cross-breeding protocol in which wild-type and knock-out neonates were exchanged at birth and fed either normal milk or milk deficient in 3′-SL or 6′-SL. At seven weeks of age, the animals were exposed to DSS for five days. Surprisingly, the St3gal4-deficient mice or wild-type mice fed with 3′-SL-deficient milk from St3gal4 knock-out mice were more resistant to DSS-induced colitis than the wild-type mice and St3gal4 knock-out mice fed with normal milk. An analysis of the gut microbiota showed different colonisation profiles depending on the presence or absence of 3′-SL in the milk. The presence of 3′-SL was associated with an enrichment in bacterial species belonging to the *Ruminococcaceae* family. The reconstitution of germ-free mice with gut microbiota isolated from St3gal4 knock-out mice demonstrated that these reconstituted mice exhibited the same sensitivity to DSS as their microbiota donor animals. Cross-breeding experiments with normal and 6′-SL-deficient milk showed no impact on the susceptibility to DSS-induced acute colitis. This elegant study clearly demonstrates the role of breastmilk oligosaccharides in shaping the intestinal flora and promoting a healthy gut immune system in adulthood. It is particularly interesting because of its experimental design, which respects the temporality and mode of the administration of breastmilk and makes it possible to study the impact of breastfeeding in adult individuals. However, sialylated oligosaccharides are not the major sugars found in human breastmilk, which contains mainly fucosylated oligosaccharides, of which 2′-fucosyl lactose (2′-FL) is the most abundant [95]. 2′-FL is not detected in mouse milk [96]. Interestingly, almost 30 years ago, a transgenic mouse model was constructed with the human gene encoding α1,2-fucosyltransferase and enabling the synthesis of 2′-FL. The expression of this gene in the mouse mammary gland promoted the significant production of 2′-FL in the milk of transgenic animals, up to a level representing 45% of the total oligosaccharides [96]. Unfortunately, to the best of our knowledge, this model has not been used to study the contribution of 2′-FL during breastfeeding on the physiology of the intestinal mucosal immunity in adulthood.

More recently, the respective role of HMOs containing fucosyl and sialyl residues on the development of gut inflammation in rodent models has been studied in a more traditional way via the oral supplementation of these oligosaccharides after weaning or in adult animals. Different models of acute or chronic colitis were used (DSS- or IL-10-deficient mice), and different doses of HMOs, alone or mixed, were administered, either preventively or curatively. It is therefore difficult to compare these different data. Nevertheless, all these studies clearly suggest that the administration of specific HMOs (mainly 2′-FL) after weaning can modify the composition of the gut microbiota in order to reduce the acute or chronic inflammation observed in the various mouse models, supporting HMO intervention as a strategy against IBD [97,98,99,100,101].

In addition to HMOs, milk also contains EVs, which are small lipid membrane vesicles that carry bioactive factors such as proteins or RNA. The oral administration of purified EVs from commercial cow’s milk for 6 days after the induction of acute colitis with DSS in C57BL/6 mice attenuated the gut inflammation and restored the gut barrier more rapidly compared to untreated animals [102]. Similar results were obtained in Balb/c mice, with a more pronounced beneficial effect of EVs purified from cow’s milk compared with those from human milk [103]. In order to assess the influence of EVs derived from cow’s milk on the composition of the gut microbiota, Zhou et al. studied two groups of mice: one fed with a diet supplemented with cow’s milk (exosome-/RNA-sufficient diet), and the other one fed with a diet supplemented with ultrasonicated cow’s milk (exosome-/RNA-depleted diet) [104]. Feeding was started at 3 weeks of age, and the intestinal content (cecum) was collected at ages 7, 15 and 47 weeks. At ages 15 and 47 weeks, the gut bacterial communities between both groups of mice turned out to be different and showed characteristics associated with certain pathologies, such as IBD, as evidenced by the decrease in the relative abundance of the *Lachnospiraceae* family in mice fed the exosome-/RNA-sufficient diet. This alteration in the gut microbiota by bovine-milk-derived EVs has been confirmed by others [105]. The same group has also recently shown and confirmed that bovine-milk-derived EVs, administered preventively, displayed a protective effect on DSS-induced colitis (acute and chronic) by suppressing intestinal inflammation and improving the gut barrier integrity [106,107]. Altogether, these results strongly suggest a beneficial immunomodulatory role for milk-derived EVs during intestinal physiology and mucosal homeostasis. However, no early conclusions should be drawn, as there are still major methodological differences between studies, particularly regarding the purification and analysis of EVs, making it impossible to compare the available data rigorously. In addition, the quantities of EVs administered are regularly supra-physiological and do not allow conclusions to be drawn about the role that they play at the doses found in breastmilk.

### 3.3. The Role of Breastfeeding in the Development of Human IBDs: Clinical Evidence

We herein propose a review of publications investigating an association between breastfeeding and the risk of developing IBD in humans (summaries of the studies can be found in Table 1 and Table 2). For this review, references published in English were obtained from a search of the PUBMED electronic database until June 2023 using combinations of the English search terms “Early nutrition”, “Early diet”, “Breastfeeding”, “Human milk”, “Inflammatory Bowel diseases”, “Crohn’s disease”, “Ulcerative colitis” and “gut health”. We identified fifty-three publications between 1979 and 2023, the majority of which relied on case–control studies (*n* = 40). Some of these studies included a broad range of predictor variables, like the environment, parental health, diet, early antibiotic usage, smoking or life-type behaviours, education and mode of delivery, that we will not be discussing in detail in this review. Most of the case–control studies analysed possible association between breastfeeding by using multivariate analysis and the diagnosis of either CD or UC as the outcome (*n* = 29): seven only had CD as the main outcome, and four only had UC as the main outcome. Five prospective cohort studies [74,108,109,110,111], seven systemic review or meta-analyses [112,113,114,115,116,117] and one recent Mendelian randomisation analysis [118] were also conducted. Among the case–control studies, nine were carried out in Asia/Pacific or Iran [119,120,121,122,123,124,125,126,127], seven in North America [128,129,130,131,132,133,134], one in Brazil [135] and twenty-two in Europe [136,137,138,139,140,141,142,143,144,145,146,147,148,149,150,151,152,153,154,155] and Israel [156,157], while one international study was conducted [158]. Thirteen case–control studies found that breastfeeding could have a marked protective effect on the development of IBD in adults [121,127,140,143,144,149] or paediatric IBD [120,125,130,148,152]. It is worth mentioning that having ever been breastfed has been associated with a differential relationship with CD or UC with a separate preventive effect [119,128,129,136,137,145,154]. Conversely, it is also commonly reported that there is no positive link between being breastfed and the occurrence of IBD [74,110,122,123,124,126,131,132,133,134,135,138,141,142,150,151,153,155,156,157,158]. Of note, it has been suggested that breastfeeding is associated with a higher risk of developing CD [139,147] or UC [144]. Overall, the literature remains inconsistent and does not support a clear association between breastfeeding and IBD. This level of great heterogeneity across studies emerged in systematic reviews [113,115,116,159] and was reported in diverse geographical areas and ethnic groups [115,159]. Concerning the latter points, it has been underlined that the magnitude of protection in individuals who were breastfed during infancy appeared higher in Asian populations compared with Caucasian people [115].

Among the case–control studies and prospective studies, 29 out of 45 analyses did consider the breastfeeding duration. Despite the considerable heterogeneity that remains in the literature regarding the interval of receiving breastfeeding, numerous studies have observed that a prolonged duration of breastfeeding could reduce the odds of having UC or CD [119,121,125,127,130,140,148,149,152,155]. Other findings have reported that a short duration of breastfeeding provides substantial protection against CD or UC [136,137,154]. Therefore, shortly after birth, breastfeeding might reduce the risk, although there is contrasting evidence that suggests that initiating breastfeeding is actually not sufficient to confer a protective effect [133]. There are population-based studies that contrast with these observations, as they did not observe associations between the length of breastfeeding and UC and/or CD diagnosis [110,124,128,129,132,136,141,142]. Few studies apart from Lopez-Serrano and Lindoso [108,145] have shown a link between exclusive breastfeeding and a change in the risk of IBD incidence [134,138,147,151] or severe illness [109]. It is worth pointing out that Lindoso et al., in their prospective study, did not reveal any association between the duration of exclusive breastfeeding and complicated disease at diagnosis [108].

Generally, the meta-analyses tended to conclude that breastfed infants are less susceptible to developing adult and paediatric-onset IBD [116,117], and that longer durations of human milk exposure increase the risk of developing IBD, although the level of evidence is low [112,113]. However, the authors acknowledged that numerous studies were of poor quality and were not strictly designed for analysing breastfeeding effects, with a lack of information on the quality and duration of breastfeeding. Failure in a proper definition of breastfeeding, the absence of a well-documented history of breastfeeding, such as inaccurate reporting of weaning, and the biased recall of whether a child was breastfed or for how long in cohort studies can lead to misinterpretations and preclude a clear conclusion of a direct link between breastfeeding and IBD. Therefore, it is still difficult to state with certainty that well-established breastfeeding prevents the onset of IBD. In fact, a spectrum of risk may cluster with breastmilk to influence early programming, including the timing of introducing different types of foods. Key variants include not only the use of bottle feeding versus exclusively breastfeeding, caesarean delivery, exposure to antibiotics or tobacco and physical activity, but also the type of IBD outcome (incidence or severity), age at diagnosis or community control design [118,159]. In addition, the paradigm that a Western lifestyle and diet [160,161] may play a key role in the development of IBD and the possibility that the strongest effect of breastfeeding on the subsequent risk of IBD was observed in Asian studies [115] fit well with the major role of the exposome in the dependent early-life effect [162]. In this case, a changing diet, socio-economic conditions of life or even improved hygiene and infection outcomes all represent relevant confounders that could underpower studies. Finally, Decker et al. pointed out that children born between 1995 and 2006 were breastfed significantly longer than children born between 1992 and 1994 [148], while Piovani et al. highlighted that the protective influence of being breastfed was higher before 2000 (OR: 0.58; 0.46–0.74) than after 2000 (OR: 0.82; 0.71–0.94) [159]. These observations have raised critical ambiguities in the overall interpretation and comparison between analyses since the 1980s in the sense that, over time, studies can differ according to the quality of the breastfeeding promotion in maternity wards and the overall improvement in the duration of breastfeeding, particularly exclusive breastfeeding.

In conclusion, despite the heterogeneity across studies, there is a trend that suggests that breastfeeding may imprint the risk of IBD. There are actually many biological plausibilities, such as microbiota development and inflammatory priming, that, under the influence of genetic predisposition [75,160], including the genetic predisposition to breastfeeding [118] or environmental exposures, make a complex interplay between breastfeeding and IBD credible.

**Table 1:** Summary of case–control/prospective studies on the association between breastfeeding and IBD.

Design	Place	Sample Size	Breastfeeding Associated with IBD	Specific Comments	Breastfeeding Duration	Main Outcome	Publication Date	Reference
Case–control study	UK	57 CD patients and 114 controls; 51 UC patients and 102 controls	Yes/No	Adults. Never breastfed was a risk factor for UC but not for CD.	No association when breastfeeding was at least 2 weeks	CD, UC	1979	Whorwell et al. [154]
Case–control study	Sweden	308 matched-pair patients and controls	Yes	Adults. There were more individuals with no or very short periods of breastfeeding among patients with Crohn’s disease than among the controls. CD overrepresented among those with no or very short periods of breastfeeding. The mean length of the breastfeeding period was 4.59 months among patients and 5.76 months among controls.	Lengths of breastfeeding collected	CD	1983	Bergstrand et al. [140]
Case–control study	International (USA, Canada, UK, Sweden, Denmark, the Netherlands, France, Italy, Israel)	302 CD patients, 197 UC patients and 998 sex- and age-matched (within 1 year) controls were studied for each patient	No	Patients whose disease started before 20 years and under study <25 years old.	Not reported	CD, UC	1987	Gilat et al. [158]
Case–control study	Canada	114 families included with one child with CD, 180 unaffected siblings as controls	Yes	Adolescents. Lack of breastfeeding was a risk factor associated with the development of CD during childhood and adolescence.	No effect of length of breastfeeding	CD	1989	Koletzko et al. [129]
Case–control study	Sweden	93 CD patients, 164 UC patients and 514 controls	No	Adults. Exclusive breastfeeding (breastfed only) or not. The comparison between cases and controls could be somewhat misleading in this study, as subsequent changes in the breastfeeding status after the mothers left the maternity ward were not recorded.	Not reported	CD, UC	1990	Ekbom et al. [138]
Case–control study	Canada	93 families included with one child with UC and 138 unaffected siblings	No	Adolescents. The lack of breastfeeding and formula feeding were not identified as risk factors during childhood.	No influence of breastfeeding duration	UC	1991	Koletzko et al. [128]
Case–control study	Sweden	167 UC patients and 167 controls	No	Adults. No difference as to how soon the patients were weaned.	Weaning < 14 days	UC	1991	Samuelsson et al. [141]
Case–control study	Sweden	152 CD patients, 135 UC patients, 305 controls	No	Adolescents and adults. Analysis did not support increased risk of IBD among individuals with no or only short durations of breastfeeding.	<2 months	CD, UC	1993	Persson et al. [142]
Case–control study	USA	68 CD patients, 39 UC patients and 202 controls	Yes	Children and adolescents. Breastfeeding was negatively associated with CD and UC, with evidence of duration-dependent trends.	≤5 months 6–11 months ≥12 months	CD, UC	1993	Rigas et al. [130]
Case–control study	USA	54 CD patients and 90 controls	No	<22 years	Not reported	CD	1996	Gruber et al. [131]
Case–control study	Italy	225 CD and 594 UC patients with age–sex-matched paired controls	Yes	Adults. Lack of breastfeeding was associated with an increased risk of CD and UC.	<4 months	CD, UC	1998	Corrao et al. [143]
Case–control study	Israel	33 CD and 55 UC patients, in 76 matched population controls and 68 clinic controls	No	Adults	Not reported	CD, UC	1998	Klein et al. [156]
Case–control study	Netherlands	290 CD patients, 398 UC patients and 616 controls	No	Adults. Breastfeeding was not associated with IBD in adults; however, a positive association was observed with pancolitis.	Not reported	CD, UC	1998	Russel et al. [146]
Case–control study	Japan	42 CD patients with 126 controls and 133 UC patients with 266 controls	Yes	<15 years. Comparison between the group fed exclusively breastmilk or mixed and the group fed by artificial (bottle) feeding alone for the development of inflammatory bowel disease. Breastfeeding during infancy until postnatal 4 months might decrease the development of chronic inflammatory bowel disease.	Not reported	CD	1999	Urashima et al. [120]
Case–control study	UK	26 CD and 29 UC patients and matched controls (eight controls for each case)	Yes	Adults. A trend for breastfed infants to have a lower risk of developing CD but a higher risk of developing UC.	Not reported	CD, UC	2000	Thompson et al. [144]
Case–control study	France	222 CD and 60 UC patients matched with controls	Yes	Before 17 years of age. Increased risk of CD development when there was exclusive or partial breastfeeding during infancy. Data not reported for UC in relation to breastfeeding.	Not reported	CD, UC	2005	Baron et al. [147]
Case–control study	Canada	194 CD patients and 194 controls	No	Less than 20 years. The proportion of case mothers who breastfed their children was similar to that of the control group.	Breastfeeding < 6 months, between 7 and 12 months, >1 year	CD	2006	Amre et al. [132]
Case–control study	China	177 UC patients and 177 age-matched and sex-matched controls	No	Adults	Not reported	UC	2007	Jiang et al. [122]
Case–control study	Germany	444 CD patients, 304 UC patients and 1481 controls	No	Adolescents (median age: 11 years old). Association between nutrition other than breastmilk at 5 months and reduced risk of both CD and UC.	Exclusive breastfeeding < 5 months versus ≥5 months	CD, UC	2007	Radon et al. [151]
Case–control study	Germany	1096 CD and 763 UC patients, 878 healthy controls	No	Adults	1 month 1–3 months 3–6 months 6 months	CD, UC	2007	Sonntag et al. [150]
Case–control study	Germany	374 CD and 169 UC patients, 743 controls	Yes	Children and young adolescents. Time of breastfeeding was not associated with CD or UC. Significantly shorter time of breastfeeding compared with the control group was found in patients with UC and CD.	The duration of breastfeeding was recorded. The average duration was 4.8 months.	CD, UC	2010	Decker et al. [148]
Case–control study	New Zealand	638 CD and 653 UC patients, 600 matched controls	Yes	Adults. Breastfeeding was protective at >3 months.	0–2 months 3–6 months 6–12 months More than 12 months	CD, UC	2010	Gearry et al. [121]
Case–control study	New Zealand	197 CD patients and 290 controls (informed about breastfeeding during infancy)	No	Age range between 5 and 86 years for the complete cohort. Being breastfed in infancy was not associated with an increased or a decreased risk of having CD.	Not reported	CD	2010	Han et al. [123]
Case–control study	Spain	124 CD patients and 235 matched controls, 146 UC patients and 278 matched controls	Yes/no	Adults. Breastfeeding, either partial or exclusive, was a protective factor for CD but not for UC in the univariate analysis.	Not reported	CD, UC	2010	Lopez-Serrano et al. [145]
Case–control study	Denmark	123 CD and 144 UC patients, 267 controls	Yes	Adults. Breastfeeding more than 6 months decreased the odds of IBD, whereas no effect of having ever been breastfed was observed.	Ever breastfed or >6 months	CD, UC	2011	Hansen et al. [149]
Prospective cohort	UK	114 CD and 66 UC patients, 248,479 controls	No	Children and early adults. Artificial versus breastfed.	Not reported	UC, CD	2011	Roberts et al. [74]
Case–control study	Iran	95 CD and 163 UC patients, 285 and 489 age- and sex-matched controls, respectively	No	Adults. No difference between breastfed infants and non-breastfed infants. No difference in mean duration of breastfeeding between IBD patients and controls (children were breastfed until almost 18 months in all groups).	Mean duration of breastfeeding reported	CD, UC	2011	Vahedi et al. [124]
Case–control study	Italy	567 CD and 428 UC patients, 562 healthy controls	No	Adults	Not reported	CD, UC	2012	Castiglione et al. [153]
Case–control study	USA	89 IBD cases and 3080 age-and membership-matched controls	No	Paediatric (<18 years). Neither exposure was associated with paediatric-onset IBD in the fully adjusted model (formula versus exclusive breastfeeding or missing).	Exclusive breastfeeding, formula feeding with or without breastfeeding or missing recorded data.	CD, UC	2012	Hutfless et al. [134]
Case–control study	Slovakia	129 CD patients, 96 UC patients, 293 controls	No	Adults. Risk of CD and UC associated with breastfeeding < 6 months.	0–5 months 6–12 months More than 12 months	CD, UC	2013	Hlavaty et al. [155]
Case–control study	Denmark	59 CD and 56 UC patients, 477 healthy controls	Yes	Children < 15 years. Breastfeeding more than 3 months was associated with a reduced risk of IBD.	>3 months as a variable in a multivariate analysis.	CD, UC	2013	Jakobsen et al. [152]
Prospective cohort	USA	146,681 248 incident cases of CD and 304 incident cases of UC	No	Adult women. No association with breastfeeding duration.	≤3 months 4–8 months ≥9 months	UC, CD	2013	Khalili et al. [110]
Case–control study	China	1308 UC patients and matched controls	No	Adults	Not reported	UC	2013	Wang et al. [126]
Prospective cohort	USA	333 CD and 270 UC patients	Yes/No	Adult patients. Breastfeeding was statistically significant in its inverse relationships with CD-related surgery; no association with UC-related surgery.	Not reported	UC, CD (IBD-related surgery)	2014	Guo et al. [109]
Case–control study	Australia	154 MEM (Middle Eastern migrant in Australia) cases (75 CD patients; 79 UC patients), 153 MEM controls, 162 Caucasian cases (85 CD patients; 77 UC patients), 173 Caucasian controls, 153 controls in Lebanon	Yes	Adults. Declined risk of CD if breastfeeding ≥ 3 months and decreased risk of UC if breastfeeding ≥ 6 months.	Breastfeeding duration effects investigated	CD, UC	2015	Ko et al. [127]
Case–control study	Asia-Pacific (China, Hong Kong, Indonesia, Sri Lanka, Macau, Malaysia, Singapore, Thailand and Australia)	442 cases and 940 controls	Yes	Childhood. Breastfeeding > 12 months reduced the risk of IBD.	0–6 months 7–12 months More than 12 months	CD, UC	2015	Ng et al. [125]
Case–control study	Canada	973 CD and 698 UC patients, 10,488 controls	No	Childhood and adolescence between 0 and 20 years old. No association between initiating breastfeeding at the time of birth or, alternatively, not initiating breastfeeding and being diagnosed with IBD later in life. The authors could not know how long breastfeeding was maintained after discharge.	Not reported	CD, UC	2016	Bernstein et al. [133]
Prospective cohort	Australia	81 CD and 51 UC patients, 103 controls	No	Adults	Not reported	CD, UC	2016	Niewiadomski et al. [111]
Case–control study	Brazil	145 CD patients and 163 controls	No	Adults	Not reported	CD	2017	Salgado et al. [135]
Case–control study	Italy	102 CD and 162 UC patients, 103 controls	Yes/No	From early childhood to adolescence (between 1 and 18 years). No association reported between breastfeeding and UC. Breastfeeding > 3 months was associated with higher risk of developing CD.	Breastfeeding > 3 months (as a variable in the multivariate analysis).	CD, UC	2017	Strisciuglio et al. [139]
Prospective cohort	North America (USA and Canada)	1119 patients with CD	Yes	Paediatric cohort. Exclusive breastfeeding inversely correlated with complicated paediatric CD. No difference according to exclusive breastfeeding duration (dichotomised < 3 months to >3 months).	Breastfeeding exposure was initially analysed as any duration of exclusive breastfeeding (of these breastfed patients, 104 (13.4%) were exclusively breastfed for less than 1 month, 170 (21.8%) for 1–3 months, 170 (21.8%) for 3–6 months and 302 (38.8%)). Subsequent analysis stratified by duration of breastfeeding and compared with never breastfed, those with 1–3 months of exclusive breastfeeding, and children with >3 months of exclusive breastfeeding.	Complicated CD, need for CD-related hospitalisation and surgery	2018	Lindoso et al. [108]
Case–control study	Switzerland	617 CD patients, 494 UC patients and 352 controls	Yes/No	Adults. No association with the risk of IBD or CD. A shorter duration (<6 months) was protective for UC.	<6 months vs. 6 months	CD, UC	2020	Lautenschlager et al. [136]
Case–control study	Netherlands	323 CD and 321 UC patients, 1348 controls	Yes/no	Adults. A protective effect was described when breastfeeding < 3 months for CD but not for UC.	<3 months vs. >3 months	CD, UC	2020	Van der Sloot et al. [162]
Case–control study	Southeast Asia (Malaysia)	38 CD and 32 UC patients, 140 healthy controls matched by gender, age and ethnicity	Yes/No	Children/adolescents (<18 years). Breastfed ≥ 6 months was protective for UC but not CD.	Duration of breastfeeding considered	CD, UC	2022	Lee et al. [119]
Case–control study	Israel	405 CD and 341 UC patients, 2043 controls	No	Adults in a population with a follow-up of 50 years.	Not reported	CD, UC	2022	Velosa et al. [157]

**Table 2:** Summary of published reviews and meta-analyses on the association between breastfeeding and IBD.

Design	Place	Sample Size	Breastfeeding Associated with IBD	Specific Comments	Breastfeeding Duration	Main Outcome	Publication Date	Reference
Meta-analysis	International	A total of 17 published studies, 5 of which were graded to be of high quality	Yes	This meta-analysis demonstrates that breastfeeding has a statistically significant protective role against UC and an even greater role against CD.	Duration of breastfeeding was sought and documented	UC, CD	2004	Klement et al. [117]
Systematic review	International	Seven studies that included patients with early-onset IBD	Yes	Breastmilk exposure had a significant protective effect against developing early-onset IBD. A non-significant difference was demonstrated for ulcerative colitis and Crohn’s disease individually.	Not reported	IBD	2009	Barclay et al. [116]
Meta-analysis	International	A total of 35 studies including 7536 patients with CD, 7353 patients with UC and 330,222 controls	Yes	Magnitude of protection higher in Asian population. Similar magnitude of lower susceptibility in paediatric and adult-onset disease.	Stronger decreased risk when breastfeeding > 12 months as compared with 3 or 6 months.	UC, CD	2017	Xu et al. [115]
Systematic review	China	Totals of 8 full texts with epidemiological data, 25 with risk factor data in Chinese and 7 full texts with epidemiological data and 12 with risk factor data in English were included for analysis.	Yes	Two references underlined a protective effect in China for UC. Not reported for CD.	Not reported	IBD	2018	Cui et al. [114]
Systematic review	International	A total of 2 of the 17 articles included for the infant milk-feeding practices and IBD examined shorter versus longer durations of exclusive human milk feeding, and none examined the intensity, proportion, or amount of human milk fed to mixed-fed infants. Thirteen articles examined the relationship between never fed human milk versus having ever been fed human milk and IBD. Nine articles examined the relationship between shorter versus longer durations of any human milk feeding and IBD.	Yes/No	The relationship between never breastfed versus having ever been breastfed human milk and the IBD risk was inconclusive. This review includes two articles, which provided insufficient evidence to draw conclusions about the relationship between the duration of exclusive breastfeeding and IBD. Having been fed human milk for short durations or not at all is associated with a higher risk of diagnosed IBD.	Shorter versus longer durations of any human milk feeding are associated with a higher risk of IBD.	IBD	2019	Güngor et al. [113]
Umbrella review of meta-analyses	International	A total of 53 eligible publications included with 71 reported risk factors for IBD.	Yes	Longer exposures were associated with decreased risk. The protective effect was greater in Asian than in Caucasian individuals (and in studies conducted before 2000).	Discussed	UC, CD	2019	Piovani et al. [159]
Meta-analysis	International	Two cohort studies and forty case–control studies.	Yes	Breastfeeding, especially of longer durations, was protective against IBD development.	Discussed	UC, CD	2021	Agrawal et al. [112]
Mendelian randomisation analysis	Europe	458,109 participants	Yes	Relationships between colitis and both physical activity and breastfeeding; breastfeeding decreased the risk of CD (in the univariate models) and UC (in the multivariate model). Genetically predicted breastfeeding was associated with lower risk of UC and CD.	Not reported	UC, CD	2023	Saadh et al. [118]

## 4. Early Determinants of Microbiota and Colitis Trajectories

### 4.1. General

It is now well established that the gut microbiota is a major contributor to the pathogeneses of IBDs in adults [163]. However, in addition to the genetic determinants of IBD, the exact environmental causes of microbial dysbiosis and the timeframe of the acquisition of a pre-dysbiotic state early in life to further predispose to IBD is far from elucidated. Whether the pathogens identified in adults are inherited directly from vertical transfer from the mother or secondarily is still unclear. Consequently, the question of the maternal transmission of beneficial bacteria that are likely to colonise the infant’s gut on a long-term basis and prevent the resilience of adult intestinal homeostasis is still being debated [164]. Lastly, the inflammatory context, possibly induced by C-section compared with vaginal delivery [165], and an inappropriate diet(s) or subsequent environmental factors may both favour pathobiont colonisation and the expansion and limit abundance of symbionts.

### 4.2. Maternal IBD and Gut Microbiota

While women with IBD maintain an intestinal dysbiosis during pregnancy, characterised by an increase in gamma-*proteobacteria* and a decrease in *bacteroidetes*, babies born to these mothers with IBD show reduced diversity and lower counts of *bifidobacteria* [166]. Of note, the biomarker of gut inflammation, faecal calprotectin, assessed in IBD mothers during pregnancy and babies, was correlated to their respective gut microbiome compositions [167]. In addition, the IBD status of mothers is a predictor of higher calprotectin levels in babies. This suggests the influence of early inflammation and the role of both maternal diseases as well as maternal microbiota on the development of further dysbiotic infant gut microbiota, regardless of genetic factors. However, obviously all babies from IBD mothers will not develop IBD, and the functional redundancy among microbes may compensate for the possible lacks.

### 4.3. Gut Microbiota and IBD: A Possible Intervention?

Defining the microbial markers of dysbiosis and what constitutes a healthy microbiota in adults is already a challenge, although many bacterial genera and even species have been clearly identified as symbionts or pathobionts. Thus, attributing specific anti-inflammatory roles and functionalities of bacteria in the early-life “unstable” microbiota is quite tricky [168]. The development of the human gut microbiome, along with distinct diets, corresponds to complex and individual dynamics comprising early and late colonisers [15,169,170]. Among these species, dominant and less abundant taxa have shown overall anti-inflammatory potential, such as species from the *Bifidobacterium* and *Bacteroidetes* genera, and, to a lesser extent, *Lactobacillus* spp. In line, other anaerobic bacteria, like *Akkermansia* and *Faecalibacterium prausnitzii*, have also demonstrated regulatory functions that contribute to homeostasis and lower inflammation. In contrast, colitogenic properties have been attributed to taxa such as *Enterococcus* and *Clostridium* spp. representatives together with an abundance of *Gamma-Proteobacteria* like *E. coli* [164]. A higher occurrence of adherent-invasive *E. coli* (AIEC) has been fully demonstrated in adult IBD patients [171] as well as in paediatric CD patients [172], but, to the best of our knowledge, there is no evidence on an early asymptomatic carriage of AIEC in neonates that could influence the onset of colitis and inflammatory symptoms. The vertical transmission of AIEC was reported in mice [173], but more consistent and reliable clinical studies are actively needed. Lastly, the breastmilk route of such a possible mother-to-infant transmission, as reported for intestinal obligate anaerobic species like *Bifidobacteria*, *Bacteroides* and *Clostridia*, should be addressed in depth [174,175].

Experimental studies have clearly demonstrated that specific dietary habits have an impact on the development of the intestinal barrier and the composition of the neonatal microbiota, with a possible influence on overall health [176] and the long-term susceptibility to chronic diseases, including inflammatory colitis [177,178,179]. During the last decades, preclinical and clinical nutritional interventions have shown great potential to address IBDs by targeting adult microbiota with either prebiotics, probiotics, synbiotics or postbiotics, based on key microbial-derived metabolites [180]. For example, a promising effect of a symbiotic preparation has been shown in reducing symptoms of paediatric IBD with a mean age of 12.6 years old [181]. Only a few trials on children have reported changes in microbiota that normalise or lower some dysbiotic-associated bacterial species [182]. However, clear data in humans are scarce, as no longitudinal clinical studies address the early microbiota composition or nutritional- and microbiota-targeting interventions with further follow-up of the onset and development of IBD.

Recently, Guo and colleagues [183] reviewed the early microbial imprinting of neonates that could define and possibly modulate either resilience against or susceptibility to IBD (see also Figure 1). They finally proposed the design of “tailored interventions” based on prebiotics or probiotics, depending on the distinct mother influence types. Of note, the timing of such interventions has to be clearly defined. Indeed, the introduction of solid foods at 3 months of age, for instance, increased the short-chain fatty acids but appeared detrimental for the gut microbiota [51]. Dosing also has to be taken into account. Barone and colleagues, in attempts to decipher the role of C-section-induced dysbiosis in gut barrier dysfunction and the associated inflammation in mice, found that an excessive exposure to very diverse microbiota too early in life was harmful, sustaining the too much too early principle [165]. In line, the mechanisms involved the “weaning reaction” occurring within a specific time window to prevent susceptibility to inflammatory diseases in the adult and to promote regulatory T-cell-mediated protection [184].

## 5. Conclusions

Most of the current recommendations for pregnant women and young children do not always consider the long-term health consequences of nutrition. Implementing optimal nutrition programs from the very beginning of life is crucial to improving child development and the well-being of populations for sustainable health. In a context in which the promotion of breastfeeding is a global priority, the focus on the benefits of breastfeeding in modifying the risk of chronic non-communicable diseases is a priority for the development of preventive strategies to promote long-term health. In this review, we summarise the evidence concerning the link between breastfeeding and the reduced risk of IBD. Overall, the data remain uncertain, partly due to the considerable heterogeneity and lack of standardisation between studies. The duration of exclusive breastfeeding is probably decisive for its lasting effect on inflammatory-mediated diseases. The microbial development origin of diseases suggests that the colonisation of the microbiota regulates immune development and may program susceptibility to hyperinflammation later in life [185]. Indeed, even an early transient dysbiosis could determine a health outcome. The composition of breastmilk (i.e., the maternal microbiome or HMOs, for example), the quality of complementary feedings, the use of antibiotics and the place of residence are all variable factors that can promote or disrupt the process of a child’s gut microbiota colonisation and pathological imprinting [184,186,187,188,189]. It is therefore difficult to identify the exact role of breastfeeding and the gut microbiome in the onset of IBD. A more holistic approach is needed to examine the impact of breastfeeding on later life events. A key question is how to translate nutritional factors into biomarkers of interest, with systemic biology as a strategic tool to characterise the molecular/biological alterations leading to IBD. As such, specific improvements in our knowledge could support interventions targeting the gut microbiome, such as prebiotics, probiotics or postbiotics, that could be used to treat or prevent diseases in a precision medicine framework.
